# A new large species of *Myloplus* (Characiformes, Serrasalmidae) from the Rio Madeira basin, Brazil

**DOI:** 10.3897/zookeys.571.5983

**Published:** 2016-03-07

**Authors:** Marcelo C. Andrade, Michel Jégu, Tommaso Giarrizzo

**Affiliations:** 1 Universidade Federal do Pará, Cidade Universitária Prof. José Silveira Netto. Laboratório de Biologia Pesqueira e Manejo dos Recursos Aquáticos, Grupo de Ecologia Aquática. Avenida Perimetral, 2651, Terra Firme, 66077830. Belém, PA, Brazil; 2 Programa de Pós-Graduação em Ecologia Aquática e Pesca. Universidade Federal do Pará, Instituto de Ciências Biológicas. Cidade Universitária Prof. José Silveira Netto. Avenida Augusto Corrêa, 1, Guamá, 66075110. Belém, PA, Brazil; 3 Institut de Recherche pour le Développement, Biologie des Organismes et Ecosystèmes Aquatiques, UMR BOREA, Laboratoire d´Icthyologie, Muséum national d’Histoire naturelle, MNHN, CP26, 43 rue Cuvier, 75231 Paris Cedex 05, France; 4 Programa de Pós-Graduação em Biodiversidade e Conservação. Universidade Federal do Pará, Faculdade de Ciências Biológicas. Avenida Cel. José Porfírio, 2515, São Sebastião, 68372010. Altamira, PA, Brazil

**Keywords:** Ostariophysi, *Myleus*, rapids, pacu, freshwater fish, taxonomy

## Abstract

*Myloplus zorroi* **sp. n.** is described from the Rio Madeira Basin in Amazonia. The new species had been treated as an undescribed *Tometes* species because of the absence of a marked abdominal keel and few small spines forming its prepelvic serrae, features commonly found in the species of the *Myleus* clade of the Serrasalmidae (species of genera *Myleus*, *Mylesinus*, *Ossubtus* and *Tometes*) and also in species of *Utiaritichthys*. *Myloplus zorroi* **sp. n.** shares the following characters with its congeners and *Utiaritichthys*: molariform teeth (versus incisiform teeth in *Myleus* clade members); a labial row of premaxillary teeth separated from lingual row by an internal gap (versus absence of internal gap between premaxillary teeth rows); and an ascending process of premaxilla wide from its base to the tip (versus ascending process tapering from its base to the tip). Like other *Myloplus* species, *Myloplus zorroi* **sp. n.** differs from *Utiariticthys* by having a deeper body, approximately 60% of standard length (versus usually less than 50% of standard length). Considering all the morphological evidence, including the presence of 13–19 low spines forming the prepelvic serrae in *Myloplus zorroi* **sp. n.** versus more than 20 high spines forming a marked prepelvic keel in other species of *Mylopus*, the new species is here assigned to *Myloplus*. Comparisons of the new species with nominal species of *Myloplus*, representatives of the *Myleus* clade, and other related taxa are provided.

## Introduction

*Myloplus* Gill, 1896 comprises large Serrasalmidae fishes that can reach up to 475 mm standard length (Jégu et al. 2003). The species of this genus, commonly known as ‘pacu’ in Brazil and ‘asitau’ or ‘kumaru’ in French Guiana, are of high commercial value, particularly in the Amazon (Jégu 2003, Meunier et al. 2004). They inhabit slow-or rapid-flowing rivers and have specialized dentition for crushing seeds (Goulding 1980, Ota et al. 2013). The Serrasalmidae members are traditionally classified according to the morphology and arrangement of teeth (Ortí et al. 2008). Géry (1972) classified the species with premaxillary teeth weakly incisiform, two rows of teeth separated by an internal gap, premaxillary labial row forming a gentle arc, and symphyseal teeth always present in the subgenus *Myloplus* of the genus *Myleus* Müller & Troschel, 1844, and recognized three species: Myleus (Myloplus) asterias (Müller & Troschel, 1844), Myleus (Myloplus) rubripinnis (Müller & Troschel, 1844), and Myleus (Myloplus) knerii (Steindachner, 1881). However, Jégu and Santos (2002), in their revision of the taxonomic status of Myleus (Myloplus) knerii, distinguished this species from the former two species in having abutting premaxillary teeth rows versus premaxillary teeth rows separated by a gap. Later, Jégu et al. (2004) elevated *Myloplus* to the generic level and allocated to it the seed-eating *Myloplus asterias* and *Myloplus rubripinnis*, both of which, in addition to having two rows of premaxillary teeth that are set apart from each other, have molariform teeth, whereas the other species have incisiform teeth.

Three species are recognized within the genus *Myloplus* according to the morphological concept of Jégu et al. (2003, 2004): *Myloplus asterias*, *Myloplus rubripinnis*, and *Myloplus planquettei* Jégu, Keith & Le Bail, 2003; two additional *Mylopus* species are recognized according to the molecular phylogeny of Ortí et al. (2008): *Myloplus ternetzi* (Norman, 1929) and *Myloplus tiete* (Eigenmann & Norris, 1900). Despite being formally recognized as *Myleus*, six additional species are recognized as belonging to the genus *Myloplus* by most recent studies (e.g. Jégu and Ingenito 2007; Andrade et al. 2013; Ota et al. 2013) because they share the features considered diagnostic of the genus by Jégu et al. (2004): *Myloplus arnoldi* (Ahl, 1936), *Myloplus levis* (Eigenmann & McAtee, 1907), *Myloplus lobatus* (Valenciennes, 1850), *Myloplus rhomboidalis* (Cuvier, 1818), *Myloplus schomburgkii* (Jardine, 1841), and *Myloplus torquatus* (Kner, 1858).

From the material collected in the Rio Madeira Basin, Brazil, a previously undescribed species was identified by Camargo and Giarrizzo (2007) as a member of the genus *Tometes* Valenciennes, 1850, probably based on the very small prepelvic serrae of the specimens and because some of them have been collected in rapids, the preferred environment of *Tometes*. However, based on morphology, these specimens are assigned to *Myloplus* and described as a new species, thus bringing the total number of *Myloplus* species currently recognized to 12.

## Methods

Counts and measurements were performed as described by Jégu et al. (2003). All measurements were calculated as proportions of the standard length (SL), and the subunits of the head are presented as proportions of the head length (HL). Measurements were taken with a digital caliper to the nearest 0.1 mm. The frequency of examined specimens with a particular count is provided within parentheses after the respective count, and the values for the holotype are indicated by an asterisk. Vertebrae and supraneural counts were made from radiographs of specimens MPEG 30663, INPA 48546 and ZUEC 10776. Additional description of dentition was performed from analysis of the dissected specimen ZUEC. The osteological terminology used is that proposed by Weitzman (1962). The total number of vertebrae includes those of the Weberian apparatus, counted as four elements, and the fused PU_1_+U_1_ counted as a single bone.

The institutional abbreviations follow Andrade et al. (2016) with addition of NMNH (National Museum of Natural History, Washington, DC), and NMW (Naturhistorisches Museum Wien, Vienna).

## Taxonomy

### Myloplus zorroi sp. n.

Taxon classificationAnimaliaCharaciformesSerrasalmidae

http://zoobank.org/DE77D64E-F9F7-4361-9741-B1E69ECF570B

**Figure 1.**
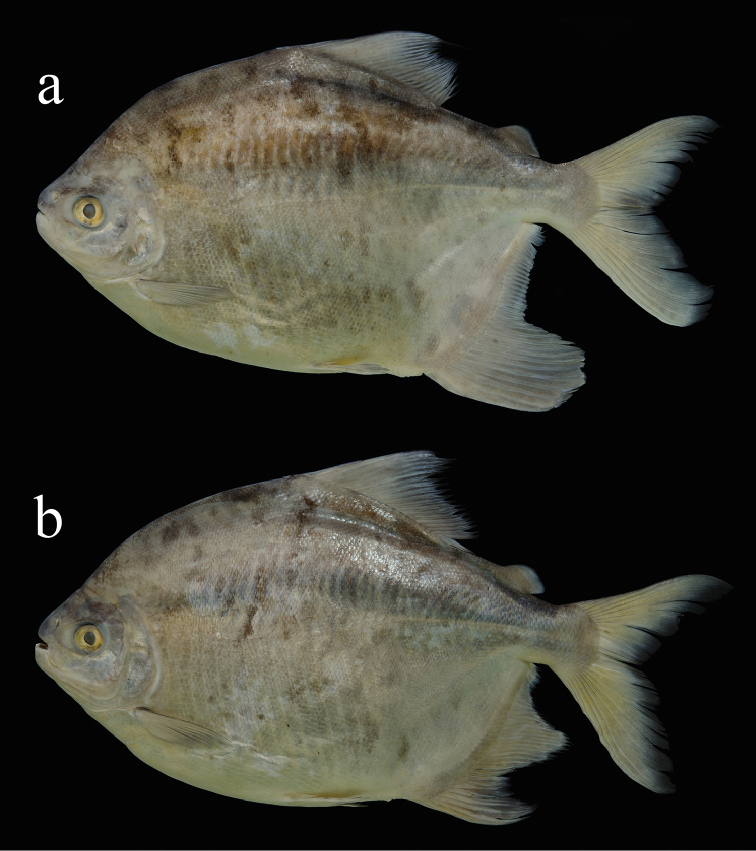
*Myloplus zorroi*, a new species from Rio Madeira Basin. **A** holotype, INPA 50880, female 326.2 mm SL **B** paratype, INPA 50868, male 339.5 mm SL. (Photographs by D. Bastos)

**Figure 2.**
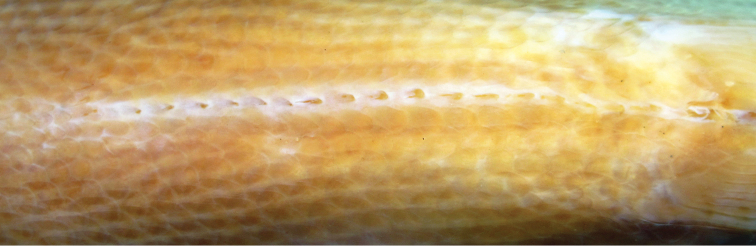
Prepelvic area of abdomen in ventral view of *Myloplus zorroi*. Paratype, MPEG 30663, male, 244.5 mm SL.

Figures 1a, b ; 2 and 4a, b, c ; Table 1

Tometes sp.: Camargo and Giarrizzo 2007: 294 [Checklist of fish species of the Marmelos Conservation Area (BX044)].

#### Holotype.

INPA 50880 (326.2 mm SL), Amazonas, Apuí, Corredeira dos Periquitos, Rio Aripuanã, 07°17'19.8"S, 60°38'10.0"W, 19 November 2014, Machado V. N. et al.

Paratypes. All from Brazil. INPA 50868 (3 specimens 183.8–339.5 mm SL), collected with holotype. MPEG 30680 (1 specimen 351.1 mm SL), Mato Grosso, Aripuanã, downstream of Salto de Dardanelos, Rio Aripuanã, 10°09'46.5"S, 59°26'54.9"W, 12 December 2014, V. Machado. MPEG 30663 (1 specimen 244.5 mm SL), INPA 48546 (1 specimen 249.9 mm SL), and ZUEC 10776, (1 specimen 246.5 mm SL), Brazil, Amazonas, Novo Aripuanã, Parque Nacional dos Campos Amazônicos, Rio Roosevelt, Madeira Basin, 8°11'51"S, 60°58'19.2"W, October 2003, M. Camargo–Zorro & T. Giarrizzo.

#### Diagnosis.

**Figure 3.**
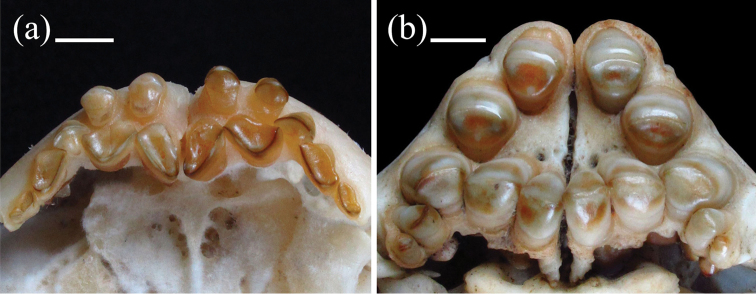
Ventral view of skeletonized premaxilla. **A** *Myloplus rubripinnis*, GEA 1301, 278 mm SL **B** *Myloplus rhomboidalis*, GEA 1500, 280 mm SL. Scale bars: 5 mm.

*Myloplus zorroi* sp. n. can be distinguished from its congeners by the absence of abdominal keel and the prepelvic serrae formed by 13–19 low spines (Fig. 2), in contrast to a well-marked abdominal keel and prepelvic serrae of more than 20 high spines. The new species is distinguished from *Myloplus asterias*, *Myloplus levis*, and *Myloplus torquatus* by the presences of fewer branched dorsal-fin rays (20–22 versus 23 or greater), and from *Myloplus arnoldi*, *Myloplus ternetzi*, and *Myloplus torquatus* by having a greater number of branched anal-fin rays (32–34 versus 31 or fewer). *Myloplus zorroi* differs significantly from *Myloplus lobatus*, *Myloplus schomburgkii*, and *Myloplus rhomboidalis* by having two rows of premaxillary teeth forming a slight arc (e.g., Fig. 3a) versus two rows of premaxillary teeth forming a shape that resembles the uppercase letter “A” (Fig. 3b). The shorter dorsal-fin base (27.6–30.1% of SL versus 31.8% of SL or higher), and the larger interdorsal distance (11.4–12.7% of SL versus 10.8% of SL or lower) are useful to distinguish *Myloplus zorroi* from *Myloplus asterias*, *Myloplus levis*, *Myloplus ternetzi*, and *Myloplus torquatus*. Furthermore, the new species differs from *Myloplus ternetzi* by the presence of a pair of symphyseal teeth versus their absence. The smaller vertical diameter of the eye (27.3–35.4% of HL versus 35.5% of HL or greater) separates *Myloplus zorroi* from *Myloplus arnoldi*, *Myloplus asterias*, *Myloplus levis*, *Myloplus lobatus*, and *Myloplus ternetzi*. *Myloplus zorroi* is additionally distinguished from *Myloplus arnoldi* and *Myloplus torquatus* by having a greater number of total vertebrae (40–41 versus 37 or less), by having anterior dorsal-fin rays lacking pigmentation (versus strongly dark pigmented anterior dorsal-fin rays in *Myloplus arnoldi*), and the presence of a diffuse dark band at caudal-fin distal border (versus the presence of a well-defined dark band in *Myloplus arnoldi*). The elongated fontanel with similarly sized anterior and posterior portions (versus very short posterior fontanel and rounded anterior fontanel) further distinguishes *Myloplus zorroi* from *Myloplus asterias*.

#### Description.

Morphometric data is presented in Table 1. Body laterally compressed, ovoid profile, greatest body depth at dorsal-fin origin (Fig. 1a, b). Dorsal profile of head distinctly convex from upper lip to vertical through anterior nares, nearly concave or gently straight from that point to distal margin of supraoccipital spine, and distinctly convex from that point to dorsal-fin origin. Dorsal-fin base slightly convex. Profile straight from dorsal-fin terminus to adipose-fin origin. Ventral profile of head slightly concave; ventral profile of body distinctly convex. Caudal peduncle relatively short, profile of lower caudal peduncle slightly concave. Anal-fin base distinctly convex at its basal third.

**Table 1.** Morphometric data of *Myloplus zorroi* (N = 8). Range values include the holotype. SD = standard deviation.

	Holotype	Range	Mean	SD
Standard length (mm)	326.2	183.8–351.1	282.9	*
		Percentages of standard length		
Body depth	59.3	59.1–62.5	60.4	1.3
Head length	24.3	21.8–24.6	23.4	0.9
Supraoccipital process	26.0	25.9–29.6	27.8	1.3
Predorsal length	53.7	51.9–55.8	54.1	1.3
Dorsal-fin base length	29.2	27.6–30.1	29.0	0.7
Interdorsal length	12.1	11.4–12.7	12.1	0.5
Adipose-fin base length	6.7	5.6–6.7	6.1	0.4
Caudal peduncle depth	10.8	10.1–11.1	10.6	0.3
Anal-fin base length	33.6	32.3–35.3	33.7	1.0
Preanal length	77.6	72.5–79.1	76.9	1.9
Prepelvic length	58.7	54.7–59.0	57.5	1.4
Prepectoral length	24.6	23.5–25.3	24.5	0.6
Anal-pelvic distance	21.5	21.2–24.0	22.6	1.0
Pelvic-pectoral distance	36.1	32.3–36.1	34.1	1.4
Width of peduncle	5.1	3.7–5.3	4.4	0.7
Pectoral-fin length	20.4	19.1–20.9	20.2	0.6
Pelvic-fin length	15.5	13.9–16.1	15.0	0.7
First anal-fin lobe length	30.7	24.0–32.4	29.4	3.5
Second anal-fin lobe length	*	13.9–18.2	15.4	2.4
Dorsal-fin length	27.9	25.2–31.1	28.2	1.6
Distance dorsal-fin origin to anal-fin origin	64.9	62.0–65.9	64.6	1.1
Distance dorsal-fin end to anal-fin origin	49.0	46.9–49.6	48.5	0.9
Distance dorsal-fin end to anal-fin end	26.0	24.1–26.7	25.3	0.8
		Percentages of head length		
Snout length	31.2	29.3–33.7	31.4	1.4
Interorbital width	53.8	49.2–56.5	53.0	2.7
Head width	66.8	64.9–71.1	68.4	2.3
Postorbital distance	34.2	32.8–36.6	34.0	1.2
Fourth infraorbital width	16.2	14.6–19.6	16.9	1.4
Eye vertical diameter	27.4	27.3–35.4	30.9	2.9
Mouth length	17.6	14.4–18.2	16.9	1.4
Third infraorbital width	11.8	11.8–14.1	12.9	0.8
Cheek gap width	9.9	9.2–12.6	11.1	1.1
Mouth width	36.1	31.7–38.5	35.9	2.0

**Figure 4.**
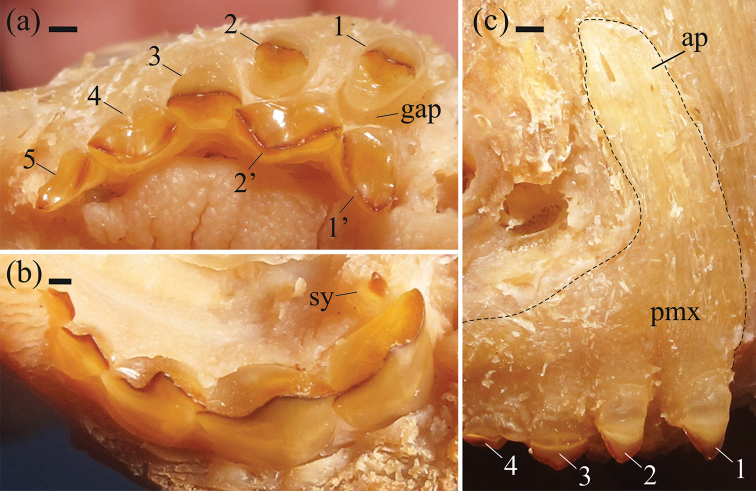
*Myloplus zorroi*, a new species from Rio Roosevelt, Madeira Basin. Paratype, ZUEC 10776, male, 246.5 mm SL. **A** Premaxilla in ventral view, molariform teeth, labial and lingual rows interspaced **B** Dentary view **C** Premaxilla in lateral view. 1–5: Labial row of premaxillary teeth; 1'–2': Lingual row of premaxillary teeth; gap: Gap between the labial and lingual rows of the premaxillary teeth; sy: Symphyseal tooth; pmx: Premaxilla; ap: Ascending process of the premaxilla. Dashed line: premaxilla contour. Scale bar: 1 mm.

**Figure 5.**
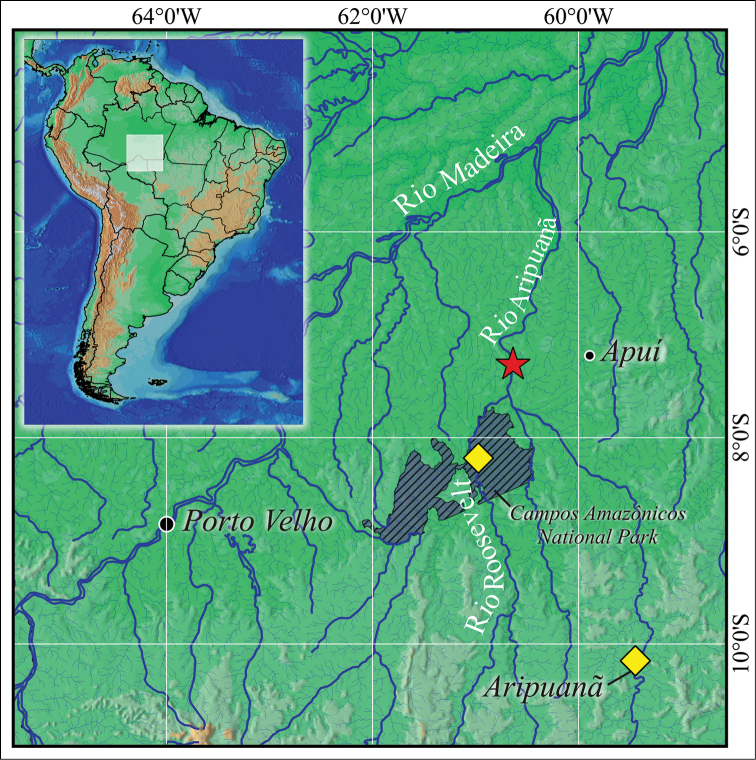
Collecting localities of *Myloplus zorroi* in Rio Madeira Basin represented by the star and diamonds, (★) type locality.

Snout gently rounded, mouth terminal, slightly oriented dorsally; jaws equal in size. Labial row of premaxillary teeth separated from lingual row by a gap; five teeth in labial row and two teeth in lingual row (Fig. 4a). Premaxillary and dentary teeth molariform. Premaxillary teeth 1–3 of labial row with sharp edges, concave in lateral view, contralateral labial series separated by distinct gap, molariform teeth 1-2 with oval base, broad anteroposteriorly, molariform tooth 3 base rounded (Fig. 4a); 4 and 5 with elongate base anteroposteriorly, distinctly concave in lateral view, and cutting edge slightly curved internally. Premaxillary teeth 1'–2' of lingual row with base somewhat trapezoidal, with cutting edge curved, and concave labial face. Dentary with 5 (2) or 6* (6) teeth, first tricuspid, 2–5 bicuspid, anterior cusp largest. Symphyseal tooth posterior to main series present. Symphyseal teeth with blade-shaped anterior margin (Fig. 4b). Maxillary edentulous. First branchial arch with gill rakers elongated, stiff, and recurved. Epibranchial gill rakers 10 (1), 11 (1), or 13 (1). Ceratobranchial gill rakers 14 (1), 15 (1), or 18 (1); one gill raker at cartilage between ceratobranchial and epibranchial.

Scales cycloid, lateral line with 80 (1), 81 (3), 82 (2), or 83* (2) perforated imbricate scales from supracleithrum to hypural joint; total perforated scales 85 (1), 86 (2), 87 (1), 88* (2), or 89 (2). Scale rows between dorsal-fin origin and lateral line 39 (1), 40* (3), 41 (2), or 42 (2). Scale rows between lateral line and pelvic-fin insertion 36 (2), 38* (2), 39 (2), 40 (1), or 42 (1). Circumpeduncular scales 34 (1), 35* (3), or 36 (4). Prepelvic serrae with 13 (1), 17 (2), 18 (2), or 19* (3) very reduced spines (Fig. 2), 8 (2), 9* (4), or 10 (2) simple postpelvic spines, and 5 (1), 6* (6), or 7 (1) double postpelvic spines. Total spines 28 (1), 31 (2), 33 (1), 34* (3), or 35 (1).

Pectoral-fin rays i, 16 (2), i, 17 (5), or i, 18* (1). Pelvic-fin rays i, 7* (7), or i, 8 (1). Dorsal-fin origin at midbody preceded by strong forward-directed spine. Dorsal-fin rays ii* (4), or iii (4), and 20 (3), 21* (4), or 22 (1); anteriormost rays longest. Anal-fin rays iii (7), or iv* (1), and 32 (3), 33* (2), or 34 (3). Adipose fin with sub-rectangular distal margin. Caudal fin forked into lobes of similar size.

Total vertebrae 40 (1), or 41 (2). Predorsal vertebrae 10 (3). Postdorsal vertebrae 15 (2), or 16 (1). Vertebrae through last dorsal-fin pterygiophore and first anal-fin pterygiophore 2 (1), or 3 (2). Supraneurals 6 (3). Neurocranium in lateral view high, short, triangular, with concavity at epiphyseal bar level. Ascending process of premaxilla broader from its base to tip, with slightly rounded edge (Fig. 4c). Lateral process of premaxilla after the last labial premaxillary tooth well developed, its length almost or more than three times the base length of the most posterior labial premaxillary tooth. Mesethmoid in dorsal view short, triangular, with broad base. Cranial fontanel elongated, with epiphyseal bar dividing anterior cranial fontanel and posterior cranial fontanel in equal parts. Dorsal process of supraoccipital spine very high.

*Color in alcohol*. Ground coloration silver brownish to yellowish silver, with pale hues. Darker coloration on humeral region. Overall pigmentation of head above eye somewhat darker than that of adjoining areas. Body more yellowish postero-ventrally on anal-fin region. Darker blotches, irregular in size and shape, scattered on the flanks (Fig. 1a, b) mainly in males. Dorsal, anal, and caudal fins somewhat yellowish, with distal margins darker, most conspicuous on the caudal fin. Pectoral and adipose fins overall hyaline. Pelvic fin hyaline with distal margin darker. Edge of teeth brownish (Fig. 4a–c).

*Color in life*. Based on photos of specimens collected by sport fishermen at Rio Aripuanã, *Myloplus zorroi* sp. n,. has ground coloration reddish silver, inconspicuous darker marks distributed on flanks, dorsum and head more darkened, and belly pale yellow. Dorsal, adipose, anal, and caudal fins yellowish brown.

*Sexual dimorphism*. The main secondary feature in mature males of *Myloplus zorroi* sp. n. is the additional anal-fin lobe centered on the 14^th^ branched ray (Fig. 1b). Darker and irregularly shaped blotches are present over flanks at maturity (Fig. 1a, b). Filamentous extensions on dorsal fin and stiff hooks laterally curved on anal fin found in species of *Tometes*, *Myleus*, *Mylesinus*, and other *Myloplus* species were not present in three examined males of *Myloplus zorroi* sp. n.

#### Distribution.

*Myloplus zorroi* is known from Aripuanã and Roosevelt rivers, two tributaries of the Rio Madeira basin (Fig. 5). The presence of the new species within a conservation unit was confirmed from the records for Rio Roosevelt in the area of the Campos Amazônicos National Park (formerly known as: Marmelos Conservation Area BX044), located on the boundaries of the Amazonas and Rondônia States, Brazil.

#### Habitat.

**Figure 6.**
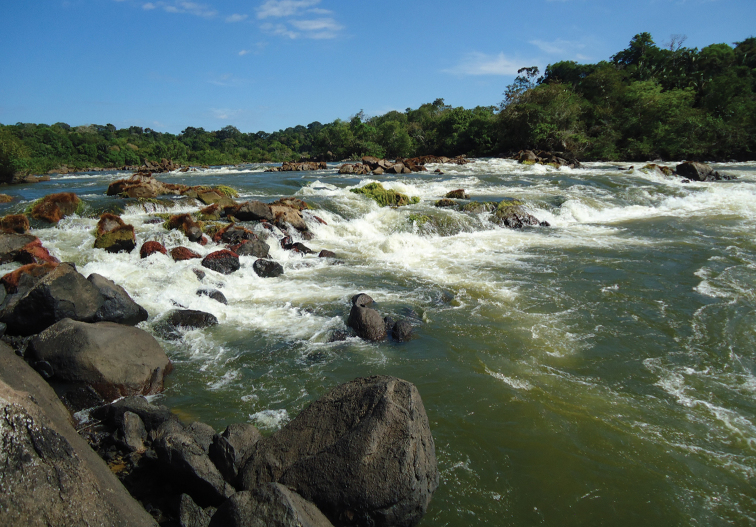
Corredeira dos Periquitos, Rio Aripuanã, type locality of *Myloplus zorroi*, Rio Madeira Basin, Brazil.

The type locality of *Myloplus zorroi* is a moderately to rapidly flowing, clear-water river running over rocky and sandy bottoms (Fig. 6), with a depth ranging from approximately 2 m to at the most 8 m, and a mean width of 320 m. The river is surrounded by extensive riparian vegetation that is mainly composed of ombrophilous forest and is located at an elevation of approximately 78 m above sea level. Water flow in the main channel is significantly reduced during the dry season (June–September), with most of the inflow restricted to small channels with rapids and extensive spread of rock outcrops scattered along the course of the main river. The records of *Myloplus zorroi* in Rio Roosevelt were collected close to the vegetated edge, while the specimens collected in Rio Aripuanã were made around the rapids of Corredeira dos Periquitos and Salto de Dardanelos.

#### Etymology.

The specific name ‘*zorroi*’ is dedicated to Mauricio Camargo-Zorro, a researcher at the Instituto Federal de Educação, Ciência e Tecnologia, in recognition of his invaluable contribution to the fish fauna inventory from the Marmelos Conservation Area. The name ‘*zorroi*’ also alludes to the Latin-American fictional character ‘Zorro’, which was the secret identity of Don Diego de la Vega, because the special features “masked” this fish as *Tometes*, concealing its true identity.

## Discussion

*Myloplus zorroi* differs markedly from its congeners in having a rounded abdominal region that lacks a marked keel and has a low number of small spines forming the prepelvic serrae (Fig. 2). This configuration is common to species of *Utiaritichthys* and the *Myleus* clade *sensu* Ortí et al. (2008), the latter mainly including representatives of the genera *Myleus*, *Mylesinus* and *Tometes*. The low number of small prepelvic spines in the *Myleus* clade was considered a derived character state by Jégu (2004: 352, character 28). The reduction of prepelvic serrae in number and size in *Myloplus zorroi* is most likely an autapomorphic character state amidst *Myloplus* species, but a phylogenetic study is needed to better characterize prepelvic features and the relationships among the species sharing them.

*Myloplus zorroi* was incorrectly classified as an unknown species of the genus *Tometes* by Camargo and Giarrizzo (2007). A more detailed analysis of its dentition and osteological features suggests that it is better classified as a species of *Myloplus*. The most important characters placing the species in *Myloplus* and not in any genera of the *Myleus* clade are as follows: molariform teeth (versus incisiform teeth); rows of premaxillary teeth separated by a gap (versus rows abutting); and ascending process of the premaxilla broad from its base to the tip, with a rounded edge that is strongly attached to the neurocranium (versus ascending process of the premaxilla narrowing from its base, with an acute edge that is weakly attached to the neurocranium). Goulding (1980) and Boujard et al. (1990) suggested that these features are possibly anatomical modifications allowing *Myloplus* specimens to crush seeds. In contrast, members of the *Myleus* clade with their incisiform teeth are specialized to cut leaves.

Several classifications of *Myloplus* have been proposed. Gill (1896) erected *Myloplus* for the South American representatives of *Myletes* Müller & Troschel, 1844 and placed the African species of *Myletes* Cuvier, 1818 in *Alestes* Müller & Troschel, 1844. Gill (1896) also proposed that *Myloplus* and *Myleus* Müller & Troschel, 1844 are subgenera of *Myleus*. Eigenmann (1915) resurrected *Myloplus*, distinguishing it from *Myleus* by features corresponding to sexual dimorphism. Gosline (1951) considered as irrelevant the characters proposed by Norman (1929) to differentiate *Myleus*, *Myloplus*, and also *Paramyloplus*, and he combined all of these genera in *Myleus*. Gosline (1951) also considered the species *Utiaritichthys sennaebragai* Miranda Ribeiro, 1937, which has reduced prepelvic serrae, as a possible species of *Myleus*. Gosline (1951) described 11 species within *Myleus*, many of which were synonymized later.

Géry (1972), following the classification of Gosline (1951), then proposed *Prosomyleus* Géry 1972 as a subgenus of *Myleus* and recognized four subgenera of *Myleus* based mainly on the arrangement and shape of the teeth: *Myloplus*, *Myleus*, *Paramyloplus*, and *Prosomyleus*. Based on molecular data, Ortí et al. (2008) indicated that *Myleus* *sensu* Géry (1972) forms a paraphyletic group and suggested that species of *Myleus* Müller & Troschel, 1844 are more closely related to *Mylesinus* and *Tometes* [the latter previously identified as ‘N. gen. A’ by Ortí et al. (1996)] than to species of *Myleus* *sensu* Géry (1972) and the subgenera *Myloplus* and *Prosomyleus*. However, a more comprehensive study involving anatomical and molecular analysis is required to further explain the relationships among these Serrasalmidae genera.

Géry (1977), as well as Gosline (1951), proposed that *Utiaritichthys sennaebragai* should be treated as *Myleus* *sensu* Géry (1972) due to its reduced prepelvic serrae; however, until a study directed to solve this question is conducted, *Utiaritichthys* is considered distinct from *Myleus*. Jégu et al. (1992) redescribed the types of *Utiaritichthys sennaebragai* and showed that reports of the species made by several authors since Gosline (1951) were actually of species not belonging to *Utiaritichthys*. Additionally, Jégu et al. (1992) described *Utiaritichthys longidorsalis* Jégu, Morais & Santos, 1992 from the Rio Aripuanã, Madeira river basin. This species can be distinguished from its syntopic *Myloplus zorroi* mainly by having an elongate body, body depth usually less than 50% of SL (versus deeper body, around 60% of SL in *Myloplus zorroi*), 24–25 branched dorsal-fin rays (versus 20–22), and 26–30 spines forming the prepelvic serrae (versus 13–19). *Utiaritichthys sennaebragai* differs from *Myloplus zorroi*, as well as *Utiaritichthys longidorsalis* by possessing a deeper body and by having 9-10 spines forming the prepelvic serrae (versus 13-19). Note that the counts of 9–13 prepelvic spines for *Utiaritichthys sennaebragai* observed by Gosline (1951), Géry (1979), and Pereira and Castro (2014) are actually from specimens of the newly described *Tometes acylorhynchus* Andrade, Jégu & Giarrizzo, 2016. In the morphological phylogeny of Jégu (2004), the two species of *Utiaritichthys* form a polytomy with the *Myloplus* clade. Pending further study, *Utiaritichthys*, which shares most of the diagnostic features of *Myloplus* such as teeth morphology and arrangement of premaxillary teeth rows, remains a separate genus.

### Comparative material

*Myloplus arnoldi*: IRSNB 21.253, 1 specimen, 147.7 mm SL, Rio Xingu, Cachoeira Von Martius, Mato Grosso, Brazil. MNHN 1998-1162, 2 specimens, 147.6–154.5 mm SL, Altamira market, Brazil. *Myloplus torquatus*: NMW 56449, 1 specimen, Paralectotype, 133 mm SL, Rio Branco, Marabitanos, Brazil. NMW 56450, Lectotype, 122 mm SL, Rio Branco, Brazil. *Myloplus ternetzi*: BMNH 1926.3.2.531-532, 2 specimens, Syntypes, 157.1–163.2 mm SL, Approuague River, Maparú Rapids, French Guiana. IEPA 3548, 5 specimens, 131.7–168.2 mm SL, Amapá, Brazil. IEPA 3560, 5 specimens, 98.4–117.5 mm SL, Flota do Amapá, Rio Araguari, Amapá, Brazil. IEPA 3586, 3 specimens, 18.1–29.9 mm SL, Oiapoque, Rio Anoitaí, Amapá, Brazil. RMNH 26467, Holotype of Myleus (Paramyloplus) ternetzi goslinei, 178.3 mm SL; and RMNH 33828, 6 specimens, Paratypes of Myleus (Paramyloplus) ternetzi goslinei, 67.1–142.6 mm SL, Brokopondo, Suriname River, Suriname. *Myloplus lobatus*: BMNH 1849.11.8.32-33, 2 specimens, Syntypes, 143.6–152.6 mm SL; and BMNH 97.11.26.8, 1 specimen, 124.2 mm SL, Rio Capim, Pará, Brazil. GEA 1988, 1 specimen, 166.7 mm SL, Parque Nacional dos Campos Amazônicos, Rio Roosevelt, Madeira Basin, Brazil. MNHN 0000-5244, 1 specimen, 188.2 mm SL, Rio Amazonas, Brazil. *Myloplus rhomboidalis*: BMNH 1926.10.27.174-6, 3 specimens, 54.4–78.8 mm SL. Rio Amazonas, Monte Alegre, Brazil. GEA 1500, 1 dry skeleton, 280 mm SL, Altamira market, Xingu Basin, Brazil. GEA 1501, 1 specimen, 230.1 mm SL, Parque Nacional dos Campos Amazônicos, Rio Roosevelt, Madeira Basin, Brazil. IRSNB 20.221, 4 specimens, 66.9–95.6 mm SL, Camopi River, Polydor, French Guiana. IRSNB 20.222, 5 specimens, 68–87.9 mm SL, Oyapock River, French Guiana. MNHN 4423, 1 specimen, 150.1 mm SL, Rio Amazonas, Brazil. MNHN A-9739, 1 specimen, 128 mm SL, Essequibo River, Guyana. MNHN A-9862, 2 specimens, 138.5–140.2 mm SL, Maná River, French Guiana. *Myloplus schomburgkii*: GEA 1974, 1 dry skeleton, 135 mm SL, Rio Xingu, Brazil. GEA 1987, 1 specimen, 224 mm SL, Parque Nacional dos Campos Amazônicos, Rio Roosevelt, Madeira Basin, Brazil. *Myloplus asterias*: BMNH 1864.1.21.33, 1 specimen, 135.9 mm SL, Essequibo River, Guyana. BMNH 1900.4.2.5, 1 specimen, 237.1 mm SL, Pará State, Rio Acará, Brazil. BMNH 1971.5.10.63, 1 specimen, 182.5 mm SL; and BMNH 1971.5.10:61-62, 2 specimens, Paralectotypes, 115.3–122.8 mm SL, Essequibo River, Guyana. BMNH 1972.7.5:91-93, 3 specimens, 117.9–135.4 mm SL, Rupununi River, Wichabai, Guyana. BMNH 1982.9.24:105-107, 3 specimens, 135.5–177.1 mm SL; and BMNH 1982.9.24:83, 1 specimen, 148.6 mm SL, Xingu Basin, Brazil. GEA 1989, 1 specimen, 198.1 mm SL, Parque Nacional dos Campos Amazônicos, Rio Roosevelt, Madeira Basin, Brazil. IEPA 2869, 1 specimen, 146.5 mm SL; and IEPA 2875, 1 specimen, 147.3 mm SL; and IEPA 2890, 1 specimen, 153.9 mm SL, Amapá, Brazil. MNHN 1998-0256, 4 specimens, 144.1–152.6 mm SL, Rio Amapari and Rio Araguari, Amapá, Brazil. *Myloplus planquettei*: IEPA 3544, 6 specimens, 136.4–167.5 mm SL; and IEPA 3545, 1 specimen, 108 mm SL, Rio Jari, Amapá, Brazil. MNHN 1997-0729, 1 specimen, Paratype, 66.7 mm SL, Maná River, Saut Valentin, French Guiana. MNHN 1997-0730, Holotype, 112.8 mm SL, Maroni River, Twenke, French Guiana. MNHN 2001-1224, 1 specimen, Paratype, 139.3 mm SL, Maná River, Kawatop, Litany, French Guiana. *Myloplus rubripinnis*: BMNH 1971.5.10.64, 1 specimen, Syntype, 76.5 mm SL, Essequibo River, Guyana. GEA 1301, 1 dry skeleton, 278 mm SL, Rio Bacajá, Brazil. IRSNB 19.298, 1 specimen, 43.9 mm SL, Distrikt Marowijne, Tapanahoni River, Paloemeu Vliegveld, Suriname. IRSNB 20.223, 3 specimens, 43.7–53.1 mm SL, Camopi River, Polydor, French Guiana. IRSNB 20.224, 2 specimens, 97.3–97.6 mm SL, Oyapock River downstream Crique Adjoumba, French Guiana. MNHN 2000-0148 (ex A-9870), 3 specimens, 176.6–224.4 mm SL, Cayenne, French Guiana. MNHN A-8632, 1 stuffed specimen, 248.3 mm SL, Cayenne, French Guiana. MNHN A-9870, 1 specimen, 285.4 mm SL, Cayenne, French Guiana. MNHN A-9895, 1 specimen, 237.2 mm SL, Colombia. RMNH 33703, 1 specimen, 177 mm SL; and RMNH 33704, 1 specimen, 183.9 mm SL, Mamadam, Surinam River, above Brokopondo, Surinam. ZMA 105-565, 2 specimens, 168.7–190.6 mm SL, Saramaca River Basin, Suriname. *Utiaritichthys longidorsalis*: INPA 3638, holotype, 198.4 mm SL, Mato Grosso, Aripuanã, Rio Aripuanã. *Utiaritichthys sennaebragai*: MZUSP 100015, 3 specimens, 28.4–72.0 mm SL, Mato Grosso, Rio Juruena downstream of bridge at BR-364. GEA 1994, 1 specimen, 245.0 mm SL, Mato Grosso, São Domingos, Rio Guaporé.

## Supplementary Material

XML Treatment for Myloplus zorroi
